# USING THE ACT-OUT QUESTIONNAIRE TO INFORM ON COMMUNITY MOBILITY AND PARTICIPATION FOR PEOPLE LIVING WITH DEMENTIA

**DOI:** 10.1093/geroni/igae098.0541

**Published:** 2024-12-31

**Authors:** Isabel Margot-Cattin, Sophie Gaber

**Affiliations:** University of Applied Sciences and Arts of Western Switzerland (HES-SO), Lausanne, Vaud, Switzerland; Jönköping University, Jönköping, Uppsala Lan, Sweden

## Abstract

For people living with dementia to continue living in their current residences or “age in place”, it is essential for them to sustain their participation in daily activities and maintain mobility within their communities. Community mobility encompasses the ability to reach places where they can participate in meaningful everyday life activities. It also includes transportation modes such as walking, biking, driving, or using public transport. Thus, we developed the Participation in ACTivities and Places OUTside Home (ACT-OUT) questionnaire based on a transactional perspective and understanding of the person-environment relationship as unfolding and dynamic, embedded and situated, and enacted and emplaced. The ACT-OUT, used in a sit-down interview setting, which can be followed by mobile (walking) interviews, to enable the collection of rich data about around 25 important places that people currently visit, have abandoned, or wish to visit. A second part of the questionnaire collects information on traveling to the place (e.g., frequency, transportation, familiarity) and on activities performed there. The ACT-OUT has been used in multiple countries, including Switzerland, Sweden, the UK, and Canada, and among different populations, notably people with and without dementia. Results show that people with dementia experience a higher rate of abandonment of places and for many more places than people without dementia. It highlights the importance of addressing community mobility and how we conceptualize, design, and adapt important places to support people living with dementia to participate in places and activities that can maintain their well-being and health as they age in their communities.

